# The Long-Term Impact of COVID-19 Lockdowns in Istanbul

**DOI:** 10.3390/ijerph192114235

**Published:** 2022-10-31

**Authors:** Elçin Tan

**Affiliations:** Department of Meteorological Engineering, Aeronautics and Astronautics Faculty, Istanbul Technical University, Maslak, 34469 Istanbul, Turkey; elcin.tan@itu.edu.tr

**Keywords:** COVID-19 lockdown, particulate matter, nitrogen dioxide, ozone, sulfur dioxide

## Abstract

The World Health Organization (WHO) have set sustainability development goals to reduce diseases, deaths, and the environmental impact of cities due to air pollution. In Istanbul, although average pollutant concentrations have been on a downward trend in recent years, extreme values and their annual exceedance numbers are high based on the air quality standards of WHO and the EU. Due to COVID-19 lockdowns, statistically significant reductions in emissions were observed for short periods. However, how long the effect of the lockdowns will last is unknown. For this reason, this study aims to investigate the impact of long-term lockdowns on Istanbul’s air quality. The restriction period is approximated to the same periods of the previous years to eliminate seasonal effects. A series of paired t-tests (*p*-value < 0.05) were applied to hourly data from 12 March 2016, until 1 July 2021, when quarantines were completed at 36 air quality monitoring stations in Istanbul. The findings reveal that the average air quality of Istanbul was approximately 17% improved during the long-term lockdowns. Therefore, the restriction-related changes in emission distributions continued in the long-term period of 476 days. However, it is unknown how long this effect will continue, which will be the subject of future studies. Moreover, it was observed that the emission probability density functions changed considerably during the lockdowns compared to the years before. Accordingly, notable decreases were detected in air quality limit exceedances in terms of both excessive pollutant concentrations and frequency of occurrence, respectively, for PM_10_ (−13% and −13%), PM_2.5_ (−16% and −30%), and NO_2_ (−3% and −8%), but not for O_3_ (+200% and +540%) and SO_2_ (−10% and +2.5%).

## 1. Introduction

Air pollution is a risk factor for human health in Europe [1]. Exceeding air quality standards causes premature deaths, which is closely related to particulate matter (PMs), NO_2_, and O_3_ exposure. In contrast, excessive PM_2.5_, PM_10_, NO_2_, and SO_2_ concentrations are associated with respiratory and heart diseases. PMs are either emitted from a source or formed due to a complex reaction of air pollutants. Particles smaller than PM_10_ can harm the lungs and bloodstream. Sensitive groups and people with heart and lung disease are risk groups. PMs can also adversely affect the environment by damaging sensitive forests, acidifying lakes and streams, and affecting ecosystem diversity, as they can spread over long distances [2]. 

NO_2_ is the indicator pollutant of the reactive gas group called NO_x_, including nitrous and nitric acid. It generally occurs with fuel combustion, and source emissions are fuel-powered vehicles, power plants, and field equipment. When inhaled in high concentrations, it can damage the human respiratory system, and prolonged exposure may cause the development of asthma. Therefore, children, the elderly, and people with asthma are at greater risk. NO_2_ exposure can also harm sensitive ecosystems, and these unfavorable environmental effects might form acid rain, nutrient pollution in coastal water, and reduced visibility due to haze in the atmosphere [3]. 

Ozone occurs at two different atmospheric levels, ground level and stratosphere. Ground-level ozone has an unfavorable impact on human health and the environment as the main component of smoke. On the other hand, stratospheric ozone is not defined as an air pollutant due to its protection from the sun’s harmful ultraviolet rays [4]. Therefore, only the ozone change at ground level was examined in this study. 

SO_2_ exposes the burning of sulfur compounds built-in coal and fuel oil naturally. Its primary sources worldwide are industrial processes, domestic fuels, and thermal power plants used for heating purposes. Very few amounts are due to diesel-fired vehicles. High concentrations of SO_2_ may cause respiratory failure and reduce lung function. It can also cause the corrosion of stone buildings and other materials, damage plants, and be the source of acid rain. The atmospheric concentrations of SO_2_ are very high in cities where coal is used daily for domestic heating. The outdoor concentrations of SO_2_ are usually high in city centers and industrial areas [5].

Anthropogenic effects are one of the main reasons for air pollution. In addition to meteorological conditions, air pollution can reach threshold levels that harm human health in the city due to activities related to high populations (traffic, industrial activities, and heating). For instance, Istanbul, the metropolitan city with the highest population in Turkey, will reach 16.3 million in 2023. Istanbul’s population increase rate has been approximately 227,000 per year since 2000. In 2020, the population decreased by approximately 56,000 for the first time since 2000 and remained at approximately 15.8 million [6]. The decrease in population in Turkey might be related to the deaths due to COVID-19 and the reverse migration. The number of tourists visiting Istanbul decreased by nearly 500,000 in 2020 to 5 million.

Moreover, Istanbul is an essential air transportation hub, and while the number of transit passengers in Istanbul was around 100 million in 2009, this number decreased to 40 million by 2020. Thence, the mortality rates of cardiovascular and respiratory diseases are also associated with high exposure to PM_10_, SO_2_, and NO_2_ in Istanbul [7]. Similarly, a relationship was found between hospital admissions related to respiratory complaints and high PM_10_, PM_2.5_, and NO_2_ episodes in the megacity [8].

In addition to population density, the factors that cause air pollution are complex [9], and meteorological aspects have an essential role in their alteration [10,11,12,13,14,15,16,17,18], depending on the location. Moreover, disaggregating and quantifying the characteristics to improve air quality is challenging. On this point, pandemic restrictions, which provide a significant natural testing environment, would help us meet the air pollution challenges. With this purpose, the early studies show that air pollution has generally improved due to COVID-19 lockdowns in many countries worldwide, such as in Argentina [19], Australia [20], Austria [21], Brazil [22], China [23,24,25], France [26], Greece [27], India [28,29,30,31], Indonesia [32], Iraq [33], Israel [34], Italy [35,36], Nepal [10], Poland [37], South Korea [38], Spain [11,39,40,41], Turkey [42,43,44], the United Kingdom [45,46], and the US [47,48,49,50]. These studies were usually based on statistical comparisons of pollutants considered before, during, and after pandemic lockdowns with either a shorter time frame or few in situ observations.

Moreover, the reduction rates of pollutants vary from country to country because each country applies its own lockdown strategies. Thus, there is a gap in the literature in that we do not know the long-term effects of lockdowns and how long they would last for a city of interest, especially on the urban scale. Thus, the main goal of this study is to determine the long-term effects of lockdowns in Istanbul. For this reason, various factors were evaluated, such as station types on an urban scale, wind direction and speed, and time on different scales. Another aim of this research is to evaluate the long-term distributions of the pollutants to detect a shift in limit exceedance frequencies concerning the air quality standards of WHO and the EU. Therefore, PM_2.5_, PM_10_, NO_2_ (including NO and NO_x_), SO_2_, and O_3_ concentration levels were investigated for three 476-day phases, from 12 March 2016, until 1 July 2021, when quarantines were completed, at 36 air quality stations in Istanbul. The main findings indicate that the reduction related to the lockdown continued during the long-term period of 476 days. The distributions show different behaviors than those for the regular periods for kurtosis and the length of tails.

## 2. Data and Methods

**Figure 1:**
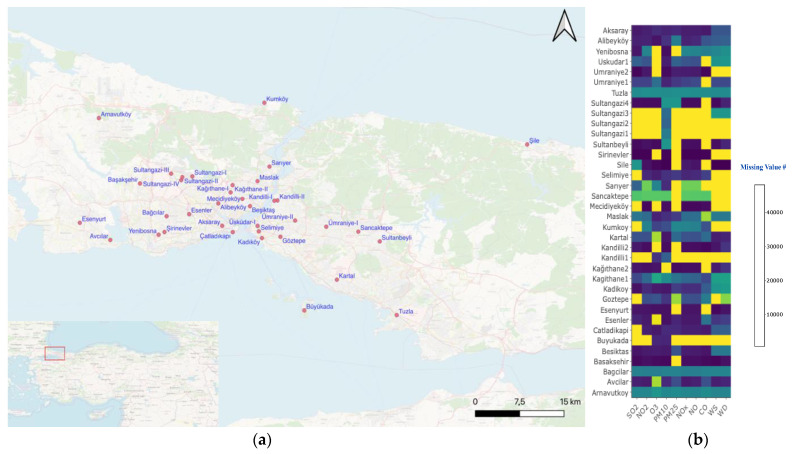
(**a**) Air quality monitoring stations in Istanbul; (**b**) missing data heatmap.

Air pollution concentration and wind data from the Istanbul Metropolitan Municipality [51] were analyzed for 36 air quality monitoring stations in Istanbul (Figure 1a). In Table 1, the types of the stations, and in Figure 1b, the missing data number of measurements as a heat map between January 2016 and June 2021 were presented. The statistically analyzed pollutants were PM_10_, PM_2.5_, O_3_, NO_2_ (including NO and NO_x_), and SO_2_. In addition, wind patterns were included in determining relations among pollutants, wind direction (WD), and speed (WS). If the missing data, shown with yellow grids in Figure 1b, were more than 30% of the total data, these stations were excluded from the study. Therefore, only the values in the grids colored blue and dark blue were included in the analysis (Figure 1b). In Istanbul, all measurements are conducted on European Union standards [51], and data quality has been checked; negative values and larger values than outliers were excluded from the analyses. A detailed description of data management is disseminated on the website of the Istanbul Municipality [51,52].

The meteorologically best available station was chosen to describe the details of the study, the statistical analyses were applied to all stations, and the results were discussed in general.

Following the WHO’s announcement of COVID-19, a global pandemic, on 11 March 2020, reasonable measures were taken in Turkey regarding the issue. Starting from 13 March 2020, schools were closed, and arrangements were made to allow people to work remotely. Due to the pandemic, the first lockdown began on 11 April 2020. This continued until June 2020 and was not applied during the summer. Afterward, between December 2020 and 1 July 2021, lockdowns, which were only valid on weekends and nights from 21:00 until 05:00, started to be implemented.

Since the lockdowns were applied at different time intervals, the analyses were carried out to cover the long-term variations and compare the differences between the time series before and during the restriction periods. In this way, the expected long-term improvement in air quality, depending on the effect of atmospheric conditions during the lockdowns, was demonstrated.

**Figure 2:**
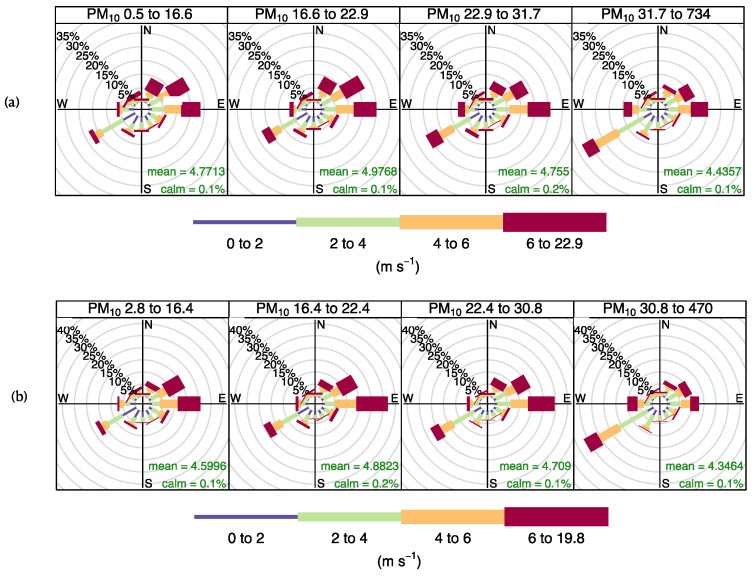
Persistence of PM_10_ observations by wind direction (%) at Sile station: (**a**) pre-lockdown period; (**b**) during-lockdown period.

Annual wind roses of the Sile station are presented in Figure 2. The wind roses were plotted for the relevant stations concerning the probability density distributions of the pollutants to analyze the wind pattern changes (not all shown here). It can be seen that the yearly differences between the pre-lockdown wind roses and the during-lockdown wind roses are negligible.

The OpenAir [53] and standard statistics packages of the R program were utilized for all statistical analysis and visualization [54]. A series of paired t-tests (*p*-value < 0.05) were applied for hourly data to determine whether there was a significant dissimilarity between the preceding lockdown period (22 November 2018–11 March 2020) (preceding-term hereafter) and the during-lockdown period (12 March 2020–30 June 2021) (Phase III hereafter), which is 476 days in total. Thus, the null hypothesis is that the mean discrepancy between before-lockdown and during-lockdown periods is zero for all pollutants, observation stations, and periods.

The number of exceedances was estimated according to the threshold values used in calculating the air quality guidelines to analyze the effect of the improvement due to lockdowns on extreme values. EU [55] and WHO standards [56] were used to calculate the exceedance numbers. Interim Targets II of WHO Air Quality Guidelines, indicated in bold, were considered valid in cases where EU and WHO standards differed in Table A1.

In order to follow the same seasonal cycle, for each pollutant, the air quality standards exceeding numbers were compared for the following 476-day periods (long-term hereafter):

- Phase I: 12 March 2016–30 June 2017 (Before Lockdowns I)
- Phase II: 12 March 2018–30 June 2019 (Before Lockdowns II)
- Phase III: 12 March 2020–30 June 2021 (During Lockdowns)

In addition, hourly, diurnal, and weekly analyses were carried out for each phase, completed in the 2-year cycle (biannual-term hereafter). For example, extended Phase I started from 30 June 2015 to 30 June 2017, in which the period split into two sub-periods called before and after, on the day lockdowns began, 12 March. Therefore, for biannual-term Phase I, the sub-period “before” is between 30 June 2015 and 11 March 2016 (256 days), whereas the sub-period “after” is 12 March 2016 and 30 June 2017, i.e., Phase I itself.

## 3. Results and Discussion

### 3.1. Particulate Matter (PMs)

Preceding-term comparisons indicate statistically significant (*p* < 0.05) differences for Istanbul’s 35 air quality stations regarding PM_2.5_ and PM_10_. For both pollutants, differences were not statistically significant for only one station, Sile, the background station located on the upwind side of the urban pollutant source. During the lockdowns, Istanbul’s air quality improved by 18% for PM_10_ and 22% for PM_2.5_, an average for the metropolis. Different rates of decrease were observed at each station depending on the station types and their distances to the pollutant source concerning the prevailing wind direction (Table 1). Annual average PM_10_ values calculated from daily averages are presented in Table 1. Most stations show annual reductions due to air pollution control from 2016 to 2020. Therefore, it is difficult to infer the recovery rates in the lockdown effect by only considering the annual averages. Although the differences between before and during the lockdowns are prominent for the vast majority of stations, it has been determined that these results cannot be evaluated reasonably because the compared periods do not include the same seasonal cycle, which is essential for the evaluation of the wind patterns [11]. However, in addition to the variability in wind direction, other meteorological factors, such as high-pressure systems [17] and Saharan dust storms [57], which cause PM_10_ to change significantly, are also affected by yearly differences. Therefore, all these meteorological factors must be excluded [16] to estimate the net effect of lockdowns on air pollution improvement. However, to quantify these factors, it is necessary to run an atmosphere–chemistry coupled dynamical model, which is not within the range of this study.

**Figure 3:**
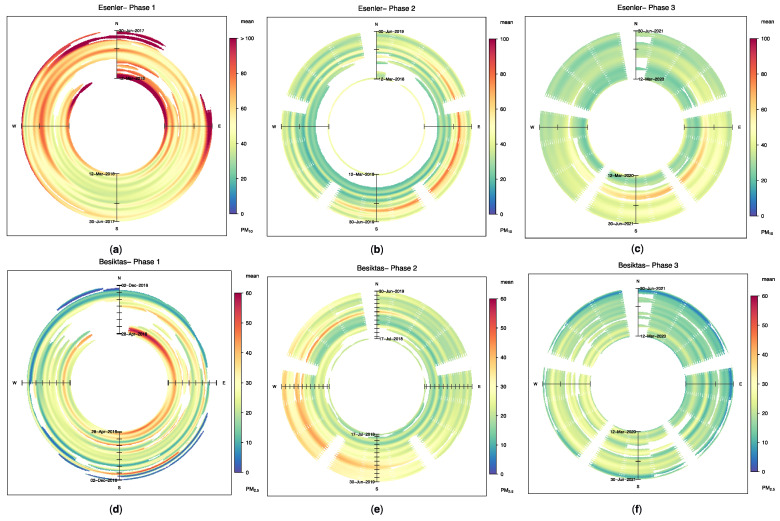
Directional trends of PM_10_ (μg/m^3^) and PM_2.5_ (μg/m^3^) for Esenler and Besiktas stations, respectively: (**a**) Esenler station PM_10_ (μg/m^3^) trends for Phase I; (**b**) Esenler station PM_10_ (μg/m^3^) trends for Phase II; (**c**) Esenler station PM_10_ (μg/m^3^) trends for Phase III; (**d**) Besiktas station PM_2.5_ (μg/m^3^) trends for Phase I; (**e**) Besiktas station PM_2.5_ (μg/m^3^) trends for Phase II; (**f**) Besiktas station PM_2.5_ (μg/m^3^) trends for Phase III.

For these reasons, the analyses through three phases, where each phase has the same seasonal cycle, were discussed as long-term analyses. In this way, it is assumed that each phase’s wind rose and seasonal factors will have slight differences that can be ignored. Following this assumption, PM_10_ (Figure 3a–c) and PM_2.5_ (Figure 3d–f) trends based on wind direction are presented for the Esenler and Besiktas stations for each phase, respectively. It can be seen that the Esenler station’s PM_10_ values decreased from Phase I to Phase II (Figure 3a,b), which are even before the lockdowns, despite the increasing population. It can be said that this drop was due to air quality control measures being applied in Istanbul. Moreover, these figures can also differentiate seasonal changes and advection effects.

On the other hand, a decrease due to the weekly and monthly lockdown effect can be seen in the northeast and southeast quadrants of Figure 3, where the pollutant sources are located, during the transition from Phase II to Phase III (Figure 3b,c). Similarly, PM_2.5_ pollutants are reduced in the Besiktas station due to the lockdowns, especially in the northwest and southwest quadrants. These are also correlated with the pollutant sources from Phase II to Phase III (Figure 3e,f).

**Figure 4:**
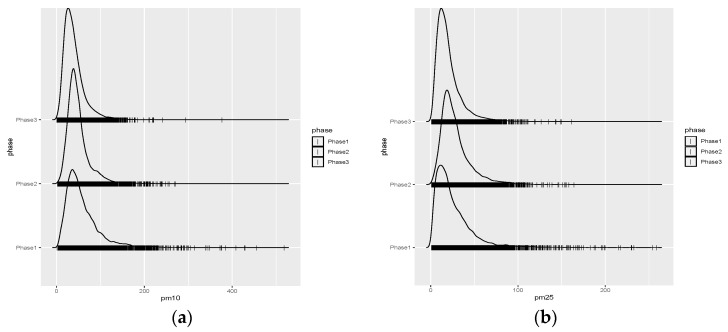
Probability density functions (PDFs) of PM_10_ (μg/m^3^) and PM_2.5_ (μg/m^3^) for Aksaray station with three Phases: (**a**) Aksaray station PM_10_ (μg/m^3^) PDFs for Phases 1–3; (**b**) Aksaray station PM_2.5_ (μg/m^3^) PDFs for Phases 1–3.

Because the atmosphere shows chaotic behavior, it can be hypothesized that the lockdown effects on the improvement in air quality can extend to longer periods from months to years. In the long term, the consequences of the lockdowns might be seen in the distribution of pollutants. Starting from this point, the variability in kurtosis revealed exceptional values for Istanbul in the long-term analysis (Phases I–III). Probability density functions (PDF) of PM_10_ and PM_2.5_ concentrations for each phase were calculated for all available stations and are presented in Figure 4 for the Aksaray station only. It can be seen that Phase III’s PDFs have shorter tails than Phases I and II. The change in the EV occurrences in the PDFs of Phase III is distinct. Although the concentrations of EV for the Aksaray station in Phase III are similar to those in other phases, their frequency of occurrence is less.

Implementing lockdowns every weekend and every evening after a particular time in Istanbul caused this recovery period to be longer. For instance, in the long-term analyses, the average value changes for Phases II and III were found to be −13% and −16% for PM_10_ and PM_2.5_, respectively (Table A2). Considering the threshold values that threaten human health, determined by the EU and WHO, significant decreases were detected in the number of values exceeding thresholds (Table A1). Limit exceedance concentrations before and during lockdown decreased by 8% for PM_10_ and 30% for PM_2.5_. Significant decreases were observed at the maximum measurements of specific stations located at the emission source regions, although the average percentage was low (Table A2).

**Figure 5:**
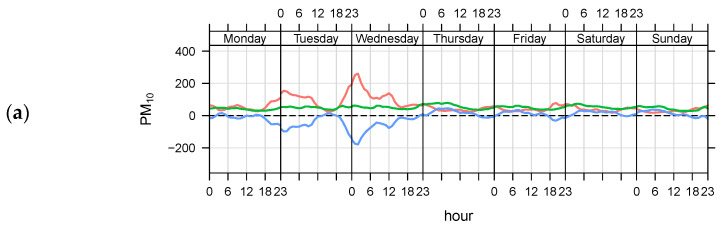

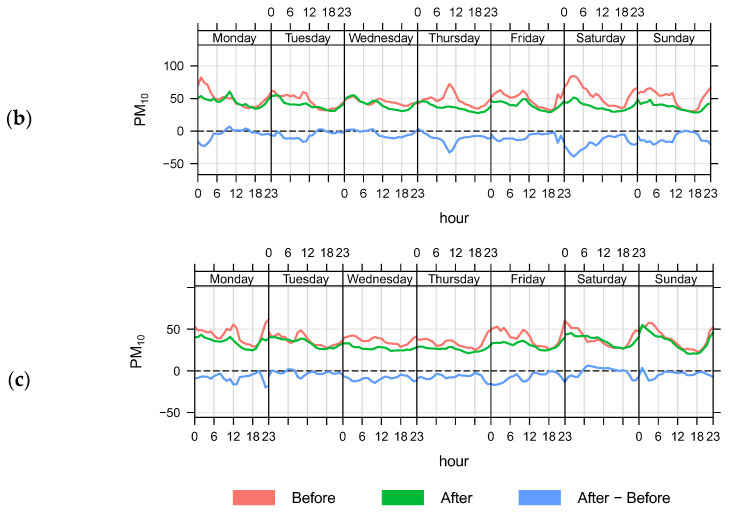
Temporal variations in PM_10_ (μg/m3) for Kadikoy station with three Phases: (**a**) Phase I differences, Before: 30 June 2015–11 March 2016 (Red), After: 12 March 2016–30 June 2017 (Green), After–Before (Blue); (**b**) Phase II differences, Before: 30 June 2017–11 March 2018 (Red), After: 12 March 2018–30 June 2019 (Green), After–Before (Blue); (**c**) Phase III differences, Before: 30 June 2019–11 March 2020 (Red), After: 12 March 2020–30 June 2021 (Green), After–Before (Blue).

The biannual-term analyses of the Phases are presented in Figure 5. The red-colored functions indicate the previous sub-period, the green-colored functions show the results during the lockdown span, and the blue-colored functions show the differences between these two periods. The thick lines of each color indicate the mean values, and the shaded extensions of the same color indicate the 95% confidence interval around the mean. According to the comparison of the Phases, the hourly and diurnal changes in PM_10_ and PM_2.5_ showed air quality improvement. In particular, the diurnal and weekly changes indicate that the lockdowns significantly helped reduce the traffic and industry-related PM_10_ and PM_2.5_ pollutants, especially for the exceedance values for each station. For example, in the Kadikoy station, for Phase III, PM_10_ hourly changes for weekdays do not exceed 75 μg/m^3^ (Figure 5c). In comparison, the maximum limit reaches 400 μg/m^3^ (Figure 4a) and 100 μg/m^3^ (Figure 5b) for Phases I and II, respectively.

Similar studies for Istanbul [42,43] show that the pollutants (PM_10_, PM_2.5_, CO, and SO_2_) decreased by 44% [42] on average in Istanbul, where rational lockdowns were implemented. The most remarkable improvements in PM_10_ and PM_2.5_ were observed in the traffic and quarry-type stations, and these results follow most similar studies [11,23].

### 3.2. Nitrogen Oxides

**Figure A1:**
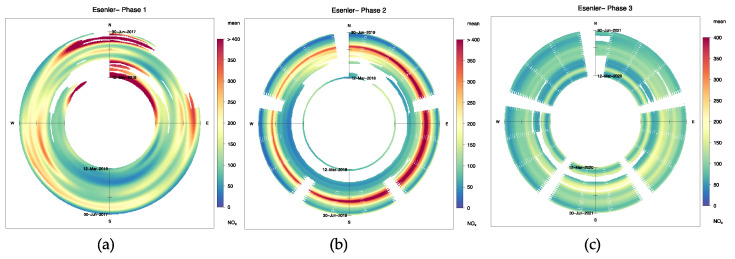
Directional trends of NO_x_ (μg/m^3^) for Esenler station: (**a**) Esenler Station NO_x_ (μg/m^3^) trends for Phase I; (**b**) Esenler Station NO_x_ (μg/m^3^) trends for Phase II; (**c**) Esenler Station NO_x_ (μg/m^3^) trends for Phase III.

**Figure A2:**
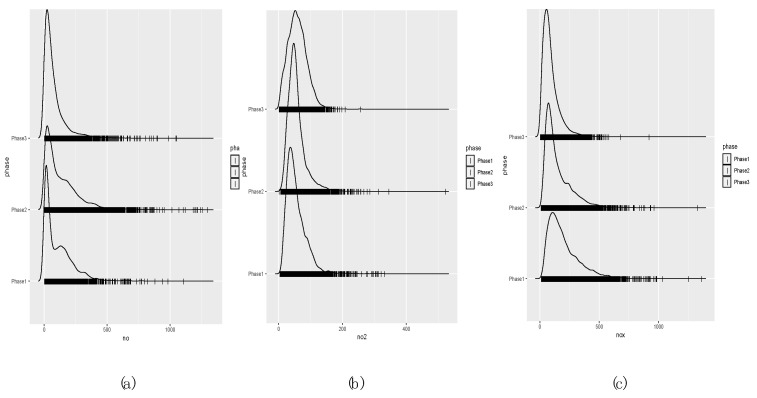
PDFs of NO (μg/m^3^), NO_2_ (μg/m^3^), and NO_x_ (μg/m^3^) (μg/m^3^) with three Phases: (**a**) Kadikoy Station NO (μg/m^3^) PDFs for Phases I–III; (**b**) Goztepe Station NO_2_ (μg/m^3^) PDFs for Phases I–III.; and (**c**) Besiktas Station NO_x_ (μg/m^3^) PDFs for Phases I–III.

In the long-term nitrogen oxide analysis, since there are no wind intensity and direction measurements at each station, directional variation graphs for only 13 stations were obtained. Although the changes due to lockdowns vary on a station basis, it was observed that the directional decreases in NO_x_ were most evident at the Esenler station (Figure A1). Regions in white in the graph indicate missing data. It can be seen from this graph that the intense pollutant source is in the east and southeast of the station (Figure A1b). In Phase III (Figure A1c), extreme NO_x_ emissions were reduced due to lockdown effects compared to Phase I and Phase II (Figure A1b and Figure A2c).

Figure A2 presents the NO, NO_2_, and NO_x_ PDFs for each of the three phases for the Kadikoy, Goztepe, and Besiktas stations, respectively. A common feature to all stations is that the PDFs of Phase II and I are similar, while those of Phase III differ. The tails of Phase III are shorter than the others, and therefore the frequency of occurrence of the extreme values is less.

**Figure A3:**
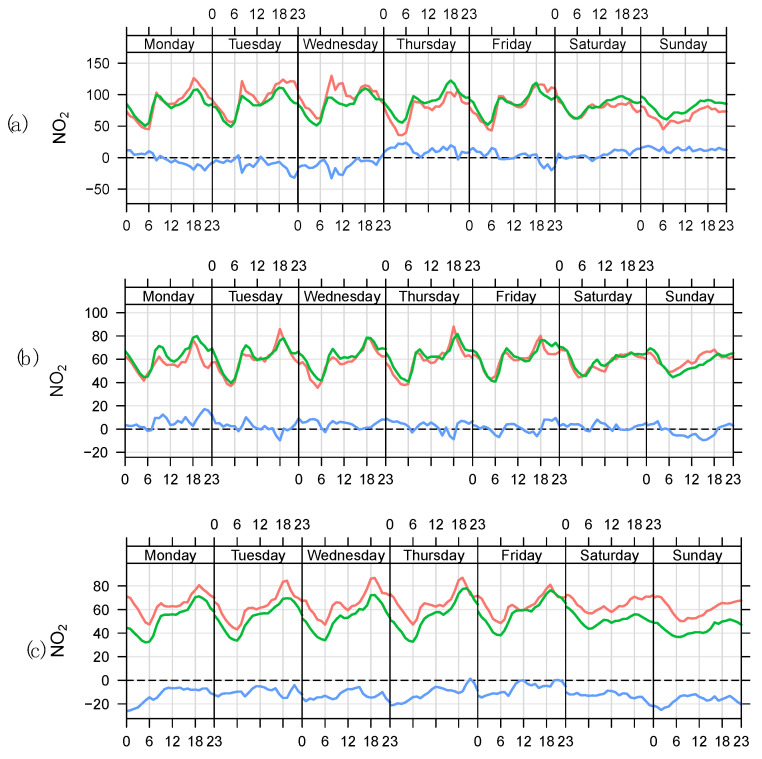
Temporal variations in NO_2_ (μg/m3) for Besiktas Station with three Phases: (**a**) Phase I differences, Before: 30 June 2015–11 March 2016 (Red), After: 12 March 2016–30 June 2017 (Green), After–Before (Blue); (**b**) Phase II differences, Before: 30 June 2017–11 March 2018 (Red), After: 12 March 2018–30 June 2019 (Green), After–Before (Blue); (**c**) Phase III differences, Before: 30 June 2019–11 March 2020 (Red), After: 12 March 2020–30 June 2021 (Green), After–Before (Blue).

The average and extreme values for this pollutant significantly change depending on the station’s location; the higher the reductions the closer the station is to the pollutant source. For example, the average value change in NO_2_ is −13% and −4% for Phases I and II with respect to Phase III, respectively, for the Esenler station (Table A2). Conversely, the air pollution exceedance increased by 10% from Phase II to Phase III (Table A3). Figure A3 presents the NO_2_ concentration changes for each Phase pivoting on the days and hours of the week. While the Phase I and Phase II changes are similar for the Besiktas station, the decreases in Phase III, where the lockdowns occur, are more prominent.

Similar to the previous studies [39], a decline in NO_x_, a lower titration effect of NO_x_, increases O_3_ due to photochemical reactions. A 10% increase in limit exceedance for NO_x_ resulted in a 540% increase in limit exceedance for O_3_.

### 3.3. Sulphur Dioxide

**Figure A4:**
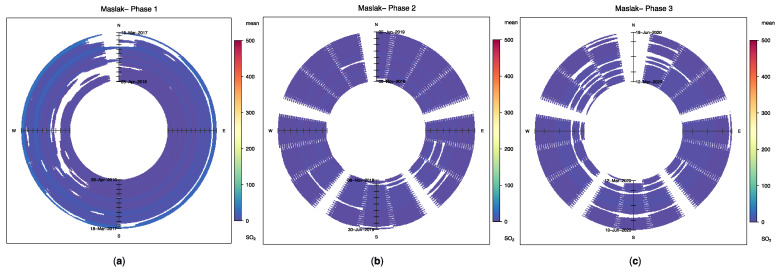
Directional trends of SO_2_ (μg/m^3^) for Maslak station: (**a**) Maslak Station SO_2_ (μg/m^3^) trends for Phase I; (**b**) Maslak Station SO_2_ (μg/m^3^) trends for Phase II; (**c**) Maslak Station SO_2_ (μg/m^3^) trends for Phase III.

The long-term analyses also demonstrate that the changes in SO_2_ due to lockdown differ by station. For example, at the Maslak station, there were no significant concentration variabilities due to wind direction from Phase II to Phase III (Figure A4). At the same time, the decrease in average values across Istanbul was determined as −9.5% between Phase II and Phase III. This decrease was −17.5% for the Kadikoy station. When the air quality standard exceedance is evaluated, Kadikoy station has a −23.5% reduction, while this value increases by 2.5% across Istanbul (Table A2 and Table A3).

**Figure A5:**
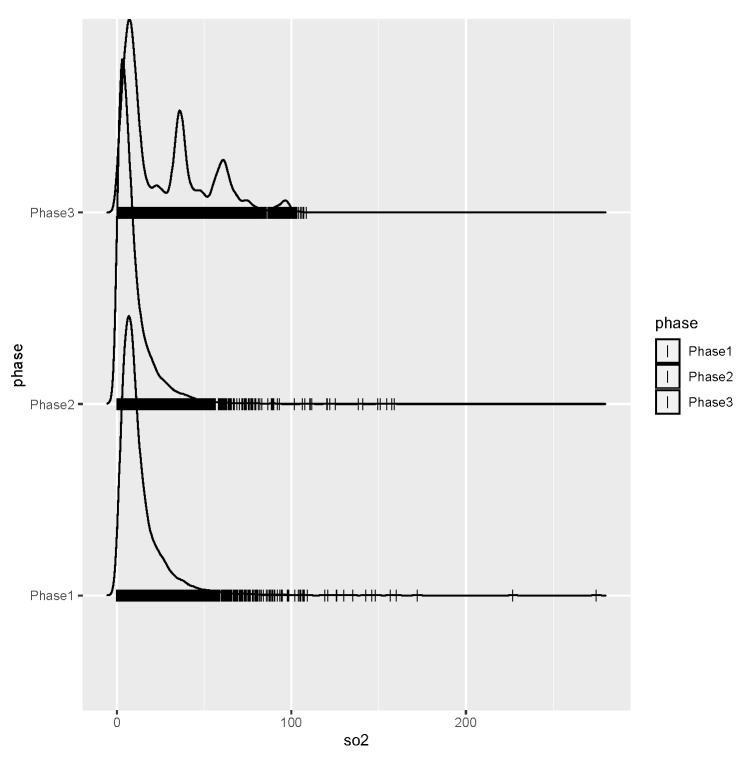
PDFs of SO_2_ (μg/m^3^) with three Phases for Kadikoy Station.

The PDF of Phase III denotes the lockdown effect based on the number of peaks and its relatively short tail for the station at Kadikoy (Figure A5). Phase III’s distribution change also shows a similar pattern with other pollutants. It is essential to note that the decrease in extreme concentration values from Phase I to Phase II in this graph of the Kadikoy station (Figure A5) is not related to lockdowns.

**Figure A6:**
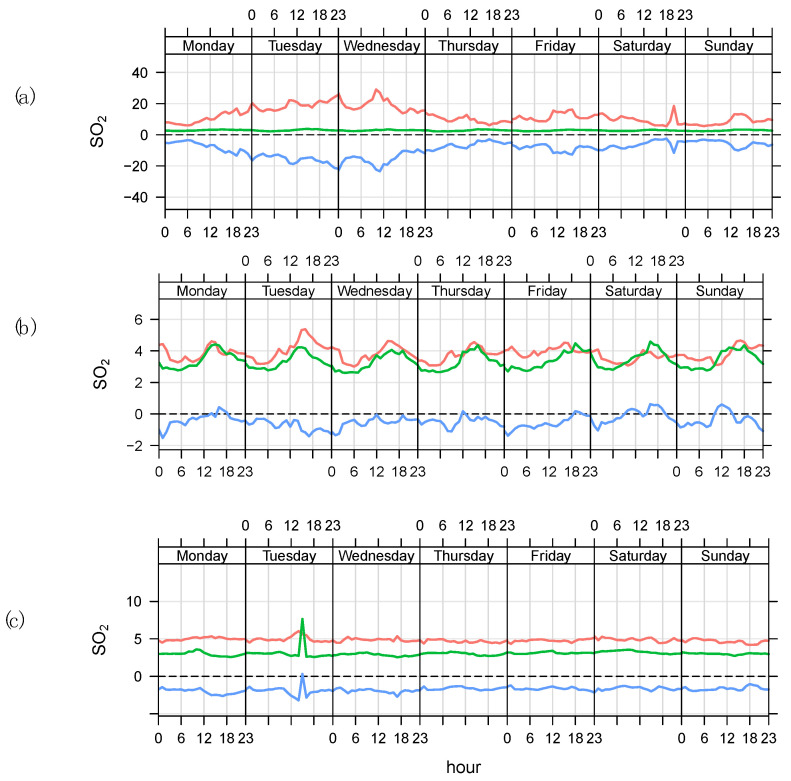
Temporal variations in SO_2_ (μg/m^3^) for Besiktas Station with three Phases: (**a**) Phase I differences, Before: 30 June 2015–11 March 2016 (Red), After: 12 March 2016–30 June 2017 (Green), After–Before (Blue); (**b**) Phase II differences, Before: 30 June 2017–11 March 2018 (Red), After: 12 March 2018–30 June 2019 (Green), After–Before (Blue); (**c**) Phase III differences, Before: 30 June 2019–11 March 2020 (Red), After: 12 March 2020–30 June 2021 (Green), After–Before (Blue).

Biannual-term comparisons show a decrease in the SO_2_ for the Besiktas station depending on the days of the week and hours (Figure A6). Long-term analysis indicates that this difference between the before (Phase II) and the during periods (Phase III) is −29.4% for the Besiktas Station (Table A2). On the other hand, the diurnal oscillation displays a similar pattern between these two phases.

Although the lockdown effect points to a decrease in average SO_2_ (−9.5%), the exceedance numbers reveal an increase (2.5%) in the long-term analyses. Similar findings were presented in Hangzhou, China [58]. The reason for this increase was related to the location of Hangzhou, which is a rural city. On the other hand, the reason that SO_2_ limit exceedance numbers were increased in Istanbul is related to the increased rates of industrial activities to compensate for the economic loss in production.

### 3.4. Ground-Level Ozone

**Figure 6:**
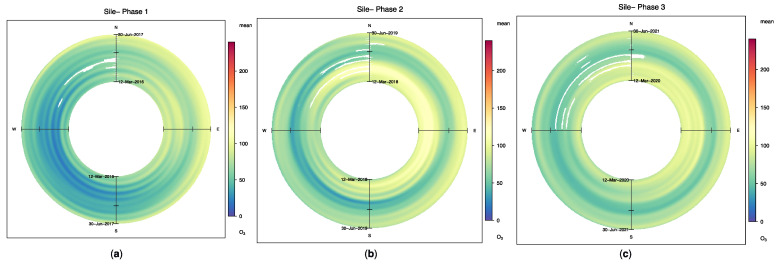
Directional trends of O_3_ (μg/m^3^) for Sile station: (**a**) Sile station O_3_ (μg/m^3^) trends for Phase I; (**b**) Sile station O_3_ (μg/m^3^) trends for Phase II; (**c**) Sile station O_3_ (μg/m^3^) trends for Phase III.

Most previous studies stated that ozone increases due to lockdowns [11,39,58]. For Istanbul, it might not be correct to end up with the same conclusion because ozone changed according to stations and periods. It was observed that the average ozone of Istanbul decreased by 8% in the comparison between Phase II and Phase III and increased by 23% in terms of Phase I and III differences. The decrease rate is similar to those of the previous studies [11]. The most prominent example of the decrease in ozone based on wind direction is at the Sile station, where Figure 6 shows that Phase III ozone emissions under the lockdown effect were less than the emission values in Phase II for almost every direction. Seasonal effects on ozone due to sunshine duration can be seen in Figure 6. As we know, change in ozone concentration is highly dependent on photochemical reactions where ultraviolet radiation, NO_x_, and VOC concentration play a significant role.

**Figure A7:**
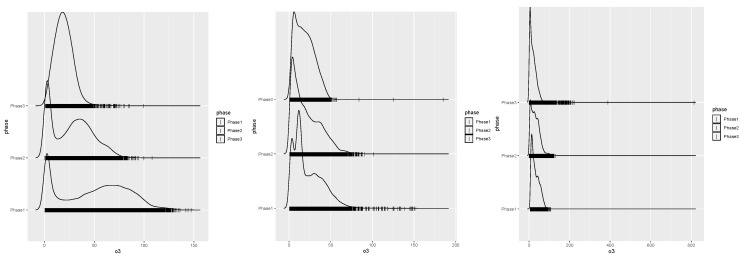
PDFs of O_3_ (μg/m^3^) with three Phases: (**a**) Maslak Station O_3_ (μg/m^3^) PDFs for Phases I–III; (**b**) Kadikoy Station O_3_ (μg/m^3^) PDFs for Phases I–III.; and (**c**) Besiktas Station O_3_ (μg/m^3^) PDFs for Phases I–III.

PDFs for ozone, as in NO_x_ emissions, show a different distribution in Phase III than in the other phases. However, the bivariate normal distribution seen in Phases I and II tends to leave its place in the standard distribution in Phase III. Although the ozone distributions of each station are different, the most prominent feature in Phase III is the modification of the tail length depending on the station. For example, the tail lengths of PDFs were shortened at the Maslak and Kadikoy stations (Figure A7a,b). In contrast, the extreme value occurrences were increased at the Besiktas station (Figure A7c) during Phase III.

**Figure A8:**
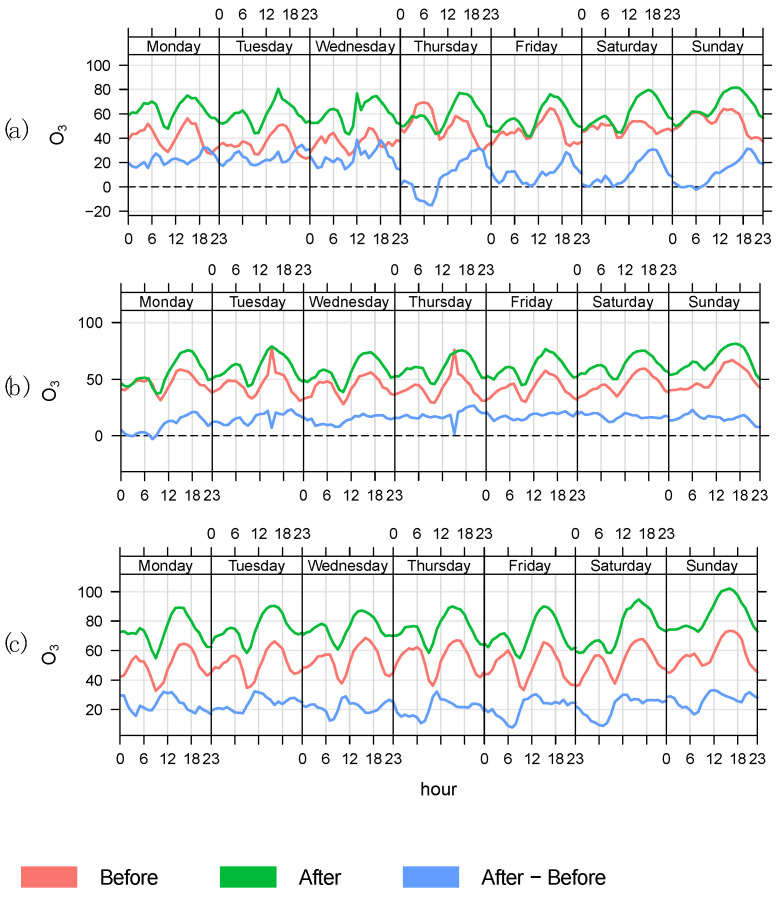
Temporal variations in O_3_ (μg/m^3^) for Basaksehir Station with three Phases: (**a**) Phase I differences, Before: 30 June 2015–11 March 2016 (Red), After: 12 March 2016–30 June 2017 (Green), After–Before (Blue); (**b**) Phase II differences, Before: 30 June 2017–11 March 2018 (Red), After: 12 March 2018–30 June 2019 (Green), After–Before (Blue); (**c**) Phase III differences, Before: 30 June 2019–11 March 2020 (Red), After: 12 March 2020–30 June 2021 (Green), After–Before (Blue).

Figure A8 presents the increase in the lockdown effect of the ozone changes at the Basaksehir station depending on the days of the week and hours. It can be observed that the daily ozone emission does not change, but the concentration of emissions increases by 20% during the transition from Phase II to Phase III.

As a result, the long-term evaluation of ozone reveals that the average ozone concentration is decreased by −8%, whereas limit exceedance numbers are increased by 540%.

### 3.5. Air Quality Limit Exceedances

**Figure 7:**
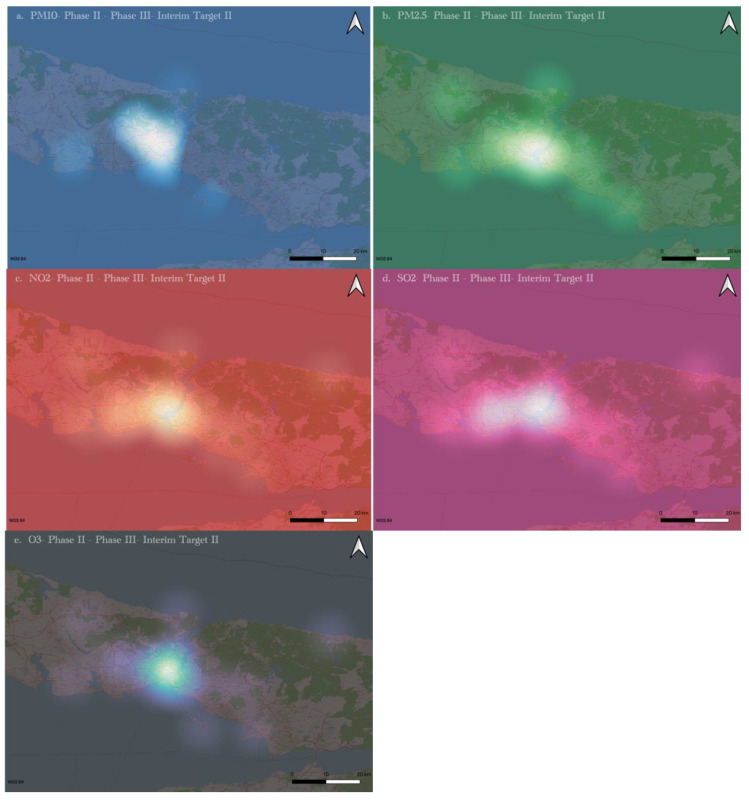
Spatial variations of Phase II and Phase III Differences in air quality exceedance: (**a**) PM_10_, (**b**) PM_2.5_, (**c**) NO_2_, (**d**) SO_2_, and (**e**) O_3_.

The spatial distribution maps of PM_10_, PM_2.5_, NO_2_, SO_2_, and O_3_ are presented in Figure 7 for the Phase II and III differences. This choice is made because the statistically significant station number is more remarkable for the comparisons of Phase II and III than for the differences between Phase I and III. The map areas close to the white color show the highest drop in the limit exceedances of pollutants due to lockdowns. Areas with light shades of these colors indicate areas with slight decreases and even increases. Dark colors indicate areas with no data. Threshold values are based on WHO Interim Target II standard values for each pollutant (Table A1). For example, in PM_10_ (Figure 7a), the decrease in the highest values indicates that the reductions in the PM_10_ values due to combustion are more dominant than the dust episodes.

Phase II and III comparisons may indicate that the change in atmospheric pollutants due to population density, related industry, and traffic activities is less than the decrease due to lockdowns. These results align with similar studies [59,60] that air pollution in Istanbul demonstrates high spatial heterogeneity.

On the other hand, in a surprising result, PM_10_ values increased in the Anatolian area of Istanbul, but not in the Bosphorus and Marmara coastal areas, during lockdowns. The fact that no decrease was observed in this region despite the lockdown may indicate the necessity of taking different measures to control air pollution. This region needs to develop more green areas [61].

Contrary to the findings of this study, previous long-term studies found that air pollution rates increase with time [62,63], possibly due to the lockdown measures being applied for less time than was the case for Istanbul. To make a significant comparison [64], we need to analyze the next “without lockdown” period as 12 March 2022–30 June 2023 (after lockdowns).

These five emission maps show that the highest decrease in emissions occurred in the south of the metropolis. Since it is known that the population is highly concentrated in this region, it is possible to say that the restrictions due to the pandemic contribute to the improvement in air quality in the regions where the population is most dense. This result also clearly shows us that the source of the air pollution in Istanbul is the same region. Therefore, the necessity of applying air pollution control measures specific to the region emerges.

## 4. Conclusions

The long-term effect of restrictions due to COVID-19 was investigated on an urban scale for Istanbul. Results indicate that the most dramatic improvements in air quality occurred in areas where human activity is most intense. That is, each district’s response to lockdown is different. In neighborhoods with already good air quality, the recovery rate due to lockdown is weak compared to neighborhoods with poor air quality. Thus, it has become clear that air quality improvement studies across Istanbul should be carried out at the scale of districts. Comparing preceding-term and long-term analyses showed that the emission decrease rates increase when we do not compare the same calendar cycles. This is most likely because other effects, such as meteorological, photochemical, or advective, cancel each other out. Therefore, we should trust same-period, long-term analyses rather than comparisons of short-term periods or periods with different cycles in Istanbul. Air quality standards are determined by the average and threshold exceedance occurrences, which can only be evaluated using long-term time series. It was found that lockdowns positively impact excessive concentration occurrences depending on the applied lockdown strategies. The distribution of the limit exceedance values varies according to the station, and it was determined that O_3_ and SO_2_ increased by 540% and 2.5% in Istanbul, respectively. On the other hand, PM_10_ (−13%), PM_2.5_ (−30%), and NO_2_ (−8%) showed a considerable decrease.

Consequently, this study leaves three critical questions for future studies that need to be solved, setting innovative strategies to sustain Istanbul’s clean air despite increasing population density: (1) How long will these long-term effects of lockdowns last for Istanbul? (2) Which lockdown strategy helped to produce the most prolonged improvements in air quality? and (3) How can we quantify the percentage of each effect separately including atmospheric stability?

Air pollution in Istanbul can be controlled by afforestation and promoting the use of electric vehicles. Moreover, lockdown-like strategies should also be considered on days when air quality thresholds are likely to be exceeded due to meteorological conditions.

## Figures and Tables

**Table 1:** Preceding-term analysis of PM_10_. Blue names indicate Anatolian Side, Green names is for stations located in the European Side.

PM_10_ (μg/m^3^)		Daily Mean					Hourly Maximum		Hourly Mean			
Station Type	Station ID and Name	2016	2017	2018	2019	2020	Before	After	Before	After	*p*-Value	Change %
Background	0134000 Kumkoy	28.1	23.9	22.4	19.0	14.3	222.2	111.9	22.4	12.0	<0.05	−46.6
	0134016 Sile	24.9	30.2	25.7	24.6	24.6	733.5	470.0	26.1	26.2	0.9691	0.2
	0134021 Buyukada	25.9	25.9	23.9	21.9	18.0	492.9	1113.0	24.0	17.2	<0.05	−28.2
Sea Traffic	0134005 Kandilli-II	42.9	40.4	35.5	34.0	28.5	985.0	202.0	37.9	29.6	<0.05	−22.0
Urban	0134011 Esenyurt	75.1	75.9	64.7	61.1	52.4	985.0	642.0	69.0	53.5	<0.05	−22.4
	0134013 Kagithane-II	-	-	-	-	-	-	-	-	-	-	-
	0134025 Yenibosna	55.6	51.8	42.6	35.3	39.7	567.3	299.9	46.1	37.9	<0.05	−17.8
	0134026 Alibeykoy	57.9	56.3	54.4	71.0	65.8	864.2	575.0	61.3	58.3	<0.05	−4.9
	0134028 Sariyer	38.0	27.3	23.9	15.8	18.7	436.6	166.2	26.0	21.8	<0.05	−15.9
	0134031 Esenler	52.1	51.1	38.4	39.1	32.8	849.5	317.0	44.3	33.3	<0.05	−24.9
	0134041 Bagcilar	-	-	-	44.2	39.9	341.3	400.1	43.2	40.0	<0.05	−7.4
	0134012 Sultanbeyli	-	-	-	33.4	32.9	143.4	140.6	35.2	32.5	<0.05	−7.4
	0134029 Kartal	48.9	57.6	50.0	47.2	39.5	707.6	706.6	49.9	37.6	<0.05	−24.7
	0134033 Kadikoy	51.9	48.1	43.9	36.6	32.2	974.0	779.6	44.3	31.7	<0.05	−28.4
	0134042 Sancaktepe	-	-	-	-	-	-	-	-	-	-	-
	0134044 Tuzla	-	-	-	45.9	34.0	625.6	468.8	43.5	41.2	<0.05	−5.3
Urban Traffic	0134014 Sultangazi-IV	-	-	-	58.5	54.7	454.5	467.6	59.1	51.1	<0.05	−13.5
	0134020 Kagithane-I	87.4	-	50.2	71.6	60.7	663.6	612.7	69.3	52.1	<0.05	−24.8
	0134036 Sultangazi-II	-	88.3	55.1	53.1	50.9	1801.4	394.5	62.6	48.6	<0.05	−22.3
	0134030 Uskudar-I	44.3	39.8	27.0	28.1	26.1	603.0	150.3	33.9	24.8	<0.05	−26.9
Rural	0134043 Arnavutkoy	-	-	36.5	34.7	41.4	344.4	238.2	35.9	39.2	<0.05	9.1
Industry Urban	0134010 Basaksehir	54.4	58.9	54.0	44.9	40.3	766.3	284.7	52.6	42.0	<0.05	−20.2
Urban Background	0134003 Avcilar	42.5	36.1	27.7	28.2	26.7	356.8	409.4	31.7	26.3	<0.05	−17.1
	0134018 Kandilli-I	-	10.5	7,8	14.7	13.0	494.2	231.9	11.2	11.6	<0.05	3.3
	0134017 Maslak	28.1	31.7	43.5	35.0	34.3	631.6	2398.4	34.6	33.0	<0.05	−4.5
	0134027 Umraniye-I	42.8	48.8	35.3	32.6	30.7	461.1	377.5	39.1	31.1	<0.05	−20.3
Quarry	0134035 Sultangazi-III	-	207.3	144.0	78.1	85.0	4213.5	1322.8	127.8	80.3	<0.05	−37.2
	0134037 Sultangazi-I	-	169.6	127.3	56.3	62.6	2540.8	823.4	104.6	64.0	<0.05	−38.9
Traffic	0134002 Catladikapi	69.0	46.5	29.7	32.2	23.0	583.2	108.7	44.2	22.6	<0.05	−48.9
	0134007 Sirinevler	23.8	57.0	48.6	45.1	42.6	398.6	338.6	43.7	41.1	<0.05	−5.9
	0134008 Mecidiyekoy	52.3	48.4	55.9	64.6	52.1	887.4	322.1	55.3	52.1	<0.05	−5.7
	0134024 Besiktas	42.0	43.2	33.5	35.3	26.6	5855.3	288.6	37.7	26.7	<0.05	−29.1
	0134032 Aksaray	68.7	59.1	51.5	47.8	40.7	633.4	376.8	55.1	39.2	<0.05	−28.9
	0134001 Selimiye	65.2	45.1	32.9	30.4	25.1	518.6	163.6	41.1	24.5	<0.05	−40.2
	0134019 Goztepe	65.7	71.1	65.7	54.4	73.0	681.8	660.4	63.0	69.0	<0.05	9.5
	0134069 Umraniye-II	56.1	39.5	40.8	29.2	32.6	668.3	380.5	41.0	36.3	<0.05	−11.3

## Data Availability

The data used and produced in the study can be requested from the author via email.

## Appendix A. Air Quality Standards of EU and WHO

**Table A1:** EU Air Quality Directives [55] and WHO Air Quality Guidelines [56].

Pollutant	Averaging Period	EU Air Quality Directives			WHO Air Quality Guidelines					
		Objective	Concentration (μg/m^3^)	Comments	Concentration (μg/m^3^)					Comments
					Interim Targets				AQG Level (μg/m^3^)	
					1	2	3	4		
PM_10_	24-h	Limit Value	50	Not to be exceeded on more than 35 days/year	150	**100**	75	50	45	**99th Percentile (i.e., 3–4 exc. days/year)**
PM_2.5_	24-h	Target Value	-		75	**50**	37.5	25	15	
NO_2_	24-h	Target Value	-		120	**50**	-	-	25	
SO_2_	24-h	Limit Value	125	Not to be exceeded on more than 3 days/year	125	**50**	-	-	40	
O_3_	8-h	Limit/Target Value	120	Max daily 8-hr mean	160	**120**	-	-	100	

## Appendix B. Average Values

**Table A2:** Long-term Average values of pollutants corresponding to the Phases and their differences.

Stations	PM_10_					PM_2.5_					NO_2_					SO_2_					O_3_				
	P I	P II	P III	III–I	III–II	P I	P II	P III	III–I	III–II	P I	P II	P III	III–I	III–II	P I	P II	P III	III–I	III–II	P I	P II	P III	III–I	III–II
	μg/m^3^			%		μg/m^3^			%		μg/m^3^			%		μg/m^3^			%		μg/m^3^			%	
Kumkoy	26.4	22.6	12.0	−54.5	−46.8	-	35.7	26.4	-	−26.0	14.8	35.2	35.7	142.2	1.6	46.3	40.5	26.9	−41.9	−33.7	16.1	55.4	56.5	250.2	1.9
Sile	26.1	24,9	26.1	−0.2	4.6	-	-	-	-	-	13.7	9.2	12.2	−10.5	32.9	40.1	38.7	45.8	14.1	18.5	80.4	92.1	86.5	7.6	−6.0
Buyukada	26.2	23.2	17.2	−34.3	−26.0	-	-	-	-	-	-	-	-	-	-	51.2	42.7	37.0	−27.6	−13.3	11.1	35.6	26.3	137.2	−26.2
Kandilli2	41.4	34.9	29.9	−27.8	−14.4	-	-	-	-	-	70.1	67.8	40.0	−43.0	−41.0	95.2	57.9	49.8	−47.7	−14.0	-	-	-	-	-
Esenyurt	72.5	63.0	54.2	−25.1	−13.9	-	-	-	-	-	38.5	57.6	25.8	−32.9	−55.2	155.1	115.2	99.4	−35.9	−13.8	55.2	47.1	31.0	−43.8	−34.2
Kagithane2	-	-	-	-	-	50.0	44.1	36.1	−27.9	−18.3	78.7	53.1	80.5	2.3	51.6	-	-	-	-	-	55.2	69.8	102.4	85.6	46.8
Yenibosna	53.6	38.6	38.1	−28.9	−1.4	-	-	-	-	-	-	80.1	72.4	-	−9.6	99.3	68.0	71.3	−28.2	4.7	-	-	-	-	-
Alibeykoy	53.8	54.9	59.2	9.9	7.7	105.5	40.2	34.0	−67.8	−15.5	103.1	61.9	62.0	−39.8	0.3	103.8	96.4	103.2	−0.5	7.1	54.2	27.0	29.0	−46.5	7.6
Sariyer	32.9	21.4	22.5	−31.6	4.7	-	-	-	-	-	-	-	29.6	-	-	58.5	41.2	40.8	−30.3	−1.0	63.6	44.9	36.5	−42.7	−18.7
Esenler	51.6	37.3	33.7	−34.7	−9.4	42.0	37.9	33.3	−20.7	−12.1	98.0	85.8	82.0	−16.3	−4.4	95.8	62.9	56.7	−40.8	−9.8	0.0	-	-	-	-
Bagcilar	-	-	-	-	-	-	48.7	41.1	-	−15.5	-	86.5	81.2	-	−6.1	-	80.4	80.9	-	0.6	-	53.0	53.9	-	1.6
Sultanbeyli	-	-	-	-	-	-	-	-	-	-	48.0	32.4	93.4	94.3	188.5	-	77.6	65.7	-	−15.4	79.0	80.0	78.5	−0.6	−1.8
Kartal	47.7	46.0	37.6	−21.3	−18.3	-	34.3	41.7	-	21.4	121.0	94.6	78.5	−35.1	−17.0	91.4	93.6	80.9	−11.4	−13.5	-	-	47.1	-	-
Kadikoy	49.2	39.6	31.8	−35.5	−19.8	71.8	39.5	33.1	−53.8	−16.1	86.6	86.4	94.3	8.9	9.1	100.0	71.8	59.3	−40.7	−17.5	31.5	29.8	25.0	−20.7	−16.0
Sancaktepe	-	-	-	-	-	-	-	-	-	-	-	-	59.0	-	-	-	-	67.2	-	-	-	-	71.0	-	-
Tuzla	-	-	-	-	-	-	34.5	22.8	-	−33.9	-	74.6	69.9	-	−6.4	-	87.4	84.0	-	−3.9	-	53.9	49.5	-	−8.1
Sultangazi4	-	-	-	-	-	-	38.5	28.7	-	−25.5	69.7	63.0	88.8	27.3	41.0	-	120.0	101.9	-	−15.0	49.7	54.9	61.3	23.4	11.7
Kagithane1	88.8	56.8	52.9	−40.5	−6.9	72.4	47.6	29.5	−59.2	−38.1	58.2	62.9	65.3	12.1	3.7	138.4	113.1	112.2	−19.0	−0.8	-	42.0	32.1	-	−23.5
Sultangazi2	89.8	55.5	48.4	−46.1	−12.8	-	-	-	-	-	-	-	-	-	-	177.6	110.2	89.4	−49.7	−18.9	-	-	-	-	-
Uskudar1	44.9	27.2	24.6	−45.1	−9.3	-	29.6	22.9	-	−22.8	76.7	80.5	57.0	−25.7	−29.3	79.9	42.9	38.3	−52.0	−10.6	-	-	-	-	-
Arnavutkoy	-	-	-	-	-	-	29.2	29.0	-	−0.7	-	44.6	41.0	-	−8.1	-	72.1	78.5	-	8.9	-	73.6	63.0	-	−14.4
Basaksehir	55.6	48.9	42.1	−24.3	−13.9	-	-	-	-	-	65.3	54.2	29.6	−54.7	−45.5	104.5	87.7	77.6	−25.8	−11.6	77.9	76.1	91.7	17.6	20.4
Avcilar	41.7	27.8	26.4	−36.8	−5.3	48.9	35.0	34.1	−30.2	−2.6	48.9	64.1	61.0	24.8	−4.9	83.2	45.7	44.9	−46.0	−1.7	-	-	47.3	-	-
Kandilli1	11.6	12.0	11.7	0.8	−2.9	-	-	-	-	-	-	-	-	-	-	27.0	24.5	27.3	1.3	11.3	15.0	35.5	30.6	103.9	−13.8
Maslak	29.0	42.8	37.3	28.6	−12.8	87.1	33.9	26.7	−69.3	−21.2	74.8	47.1	37.4	−50.0	−20.6	54.8	84.5	77.3	40.9	−8.5	63.5	34.8	26.8	−57.7	−23.0
Umraniye1	47.9	33.6	31.4	−34.5	−6.5	45.0	38.9	37.0	−17.9	−5.0	56.7	64.7	64.4	13.5	−0.4	86.5	65.2	61.7	−28.7	−5.4	76.8	54.0	28.9	−62.3	−46.4
Sultangazi3	186.1	114.9	81.5	−56.2	−29.1	-	-	-	-	-	-	-	-	-	-	382.1	237.8	154.7	−59.5	−35.0	-	-	-	-	-
Sultangazi1	141.3	101.7	64.9	−54.0	−36.1	-	-	-	-	-	-	-	-	-	-	327.8	210.9	126.1	−61.6	−40.2	-	-	-	-	-
Catladikapi	58.9	31.4	22.6	−61.7	−28.2	38.5	32.6	28.5	−25.9	−12.5	116.8	100.2	86.9	−25.6	−13.3	112.7	56.9	35.4	−68.6	−37.7	29.5	34.1	39.0	32.2	14.3
Sirinevler	36.4	47.3	41.5	14.1	−12.2	-	-	-	-	-	98.6	108.3	79.0	−19.9	−27.0	62.8	75.1	68.2	8.6	−9.2	-	-	-	-	-
Mecidiyekoy	51.5	62.3	52.6	2.1	−15.5	-	-	-	-	-	108.2	97.2	70.9	−34.5	−27.1	109.0	101.5	84.9	−22.1	−16.4	-	-	-	-	-
Besiktas	41.1	35.1	27.3	−33.6	−22.1	53.9	36.6	28.6	−46.8	−21.7	135.3	93.8	79.7	−41.1	−15.0	69.4	70.3	49.6	−28.5	−29.4	40.3	42.1	30.1	−25.3	−28.4
Aksaray	59.1	49.3	39.2	−33.6	−20.4	53.2	44.8	34.5	−35.1	−22.9	106.1	118.3	114.4	7.8	−3.3	104.9	74.3	62.5	−40.5	−15.9	28.2	28.1	24.8	−12.2	−11.7
Selimiye	55.7	31.9	24.4	−56.1	−23.5	35.4	27.0	27.8	−21.5	3.1	88.0	97.2	86.3	−1.9	−11.2	95.8	50.8	40.7	−57.5	−19.8	48.8	48.1	39.0	−20.1	−18.9
Goztepe	64.0	59.4	68.5	7.0	15.3	119.8	-	-	-	-	109.4	120.5	90.2	−17.6	−25.1	123.0	110.5	145.8	18.6	32.0	17.7	36.6	37.2	110.0	1.8
Umraniye2	51.7	36.9	37.1	−28.3	0.5	37.3	35.6	26.0	−30.3	−26.9	121.5	102.0	64.4	−47.0	−36.8	94.7	66.1	69.2	−27.0	4.6	-	-	-	-	-
Average	56.4	44.2	37.8	−27.0	−12.9	61.5	37.2	31.1	−39.0	−15.6	80.3	73.9	65.6	−6.5	−2.7	109.3	82.2	71.8	−27.9	−9.5	44.7	49.9	47.9	22.9	−8.1

## Appendix C. Exceedance Numbers

**Table A3:** Exceedance numbers of pollutants corresponding to the Phases and their differences for Interim Target II of WHO.

Stations	PM_10_					PM_2.5_					NO_2_					SO_2_					O_3_				
	P I	P II	P III	III–I	III–II	P I	P II	P III	III–I	III–II	P I	P II	P III	III–I	III–II	P I	P II	P III	III–I	III–II	P I	P II	P III	III–I	III–II
	#			%		#			%		#			%		#			%		#			%	
Kumkoy	9	12	1	−88.9	−91.7	0	74	36	38	−51.4	-	66	72	-	9.1	-	-	-	-	-	-	23	8	-	−65.2
Sile	7	7	18	157.1	157.1	-	-	-	-	-	4	6	3	−25.0	−50.0	87	72	142	63.2	97.2	40	176	73	82.5	−58.5
Buyukada	12	15	11	−8.3	−26.7	-	-	-	-	-	-	-	-	-	-	-	-	-	-	-	-	16	4	-	−75.0
Kandilli2	112	44	36	−67.9	−18.2	-	-	-	-	-	307	323	116	−62.2	−64.1	297	212	178	−40.1	−16.0	-	-	-	-	-
Esenyurt	275	182	158	−42.5	−13.2	-	-	-	-	-	108	275	23	−78.7	−91.6	452	417	398	−11.9	−4.6	10	24	0	−100	−100
Kagithane2	-	-	-	-	-	-	-	-	-	-	306	218	330	7.8	51.4	-	-	-	-	-	6	11	381	6250	3364
Yenibosna	143	67	97	−32.2	44.8	-	-	-	-	-	-	287	348	-	21.3	362	251	252	−30.4	0.4	-	-	-	-	-
Alibeykoy	133	151	145	9.0	−4.0	149	99	90	9	−9.1	413	284	280	−32.2	−1.4	267	348	332	24.3	−4.6	8	1	3	−62.5	200
Sariyer	44	7	7	−84.1	0.0	-	-	-	-	-	-	-	39	-	-	210	121	121	−42.4	0.0	2	2	5	150	150
Esenler	136	67	54	−60.3	−19.4	87	92	76	16	−17.4	400	360	396	−1.0	10.0	337	215	203	−39.8	−5.6	-	-	-	-	-
Bagcilar	0	96	116	-	-	-	153	124	29	−19.0	-	375	356	-	−5.1	-	287	291	-	1.4	0	15	4	-	−73.3
Sultanbeyli	0	57	86	-	50.9	-	-	-	-	-	211	91	279	32.2	206.6	-	137	208	-	51.8	49	124	69	40.8	−44.4
Kartal	112	135	120	7.1	−11.1	0	98	127	−29	29.6	136	339	325	139	−4.1	397	310	280	−29.5	−9.7	-	-	-	-	-
Kadikoy	109	82	62	−43.1	−24.4	121	89	68	21	−23.6	352	385	324	−8	−15.8	264	226	173	−34.5	−23.5	4	1	1	−75	0
Sancaktepe	0	0	70	-	-	-	-	-	-	-	-	-	214	-	-	-	-	213	-	-	0	0	71	-	-
Tuzla	0	110	145	-	-	-	71	14	57	−80.3	-	284	337	-	18.7	-	282	324	-	14.9	0	3	3	-	0
Sultangazi4	0	143	208	-	-	-	57	63	−6	10.5	324	307	230	−29	−25.1	-	236	410	-	73.7	3	1	50	1567	4900
Kagithane1	29	149	130	348.3	−12.8	120	136	50	86	−63.2	135	238	212	57	−10.9	54	288	214	296.3	−25.7	0	1	4	-	300
Sultangazi2	73	212	100	37	−52.8	-	-	-	-	-	-	-	-	-	-	-	-	-	-	-	-	-	-	-	-
Uskudar1	87	14	4	−95.4	−71.4	-	41	21	-	−48.8	86	345	229	166.3	−33.6	262	112	102	−61.1	−8.9	-	-	-	-	-
Arnavutkoy	0	89	123	-	-	-	62	60	-	−3.2	-	146	148	-	1.4	-	279	310	-	11.1	0	14	4	-	−71.4
Basaksehir	178	121	118	−33.7	−2.5	-	-	-	-	-	284	199	98	−65.5	−50.8	376	347	296	−21.3	−14.7	38	37	157	313.2	324.3
Avcilar	104	10	12	−88.5	20	147	61	63	−57.1	3.3	166	292	276	66.3	−5.5	293	150	149	−49.1	−0.7	-	-	-	-	-
Kandilli1	0	0	28	-	-	-	-	-	-	-	-	-	-	-	-	-	-	-	-	-	1	66	22	2100	−66.7
Maslak	39	117	86	120.5	−26.5	135	71	36	−73.3	−49.3	194	163	15	−92.3	−90.8	178	299	257	44.4	−14.0	21	0	0	−100	-
Umraniye1	115	68	52	−54.8	−23.5	117	102	90	−23.1	−11.8	155	266	185	19.4	−30.5	328	253	225	−31.4	−11.1	5	2	2	−60	0
Sultangazi3	71	322	340	378.9	5.6	-	-	-	-	-	-	-	-	-	-	-	-	-	-	-	-	-	-	-	-
Sultangazi1	55	281	199	261.8	−29.2	-	-	-	-	-	-	-	-	-	-	-	-	-	-	-	-	-	-	-	-
Catladikapi	258	23	1	−99.6	−95.7	96	44	27	−71.9	−38.6	445	434	315	−29.2	−27.4	-	-	-	-	-	0	1	1	-	0
Sirinevler	90	77	59	−34.4	−23.4	-	-	-	-	-	430	443	389	−9.5	−12.2	238	366	309	29.8	−15.6	-	-	-	-	-
Mecidiyekoy	199	187	124	−37.7	−33.7	-	-	-	-	-	475	465	416	−12.4	−10.5	-	-	-	-	-	-	-	-	-	-
Besiktas	70	34	13	−81.4	−61.8	144	86	21	−85.4	−75.6	469	417	340	−27.5	−18.5	275	271	190	−30.9	−29.9	0	1	31	-	3000
Aksaray	155	81	50	−67.7	−38.3	147	126	70	−52.4	−44.4	408	463	461	13	−0.4	341	363	263	−22.9	−27.5	43	15	0	−100	−100
Selimiye	120	25	5	−95.8	−80	63	29	21	−66.7	−27.6	385	415	374	−2.9	−9.9	-	-	-	-	-	4	1	3	−25	200
Goztepe	208	194	231	11.1	19.1	-	-	-	-	-	112	417	425	279.5	1.9	123	110.5	145.8	18.6	32	2	1	2	0	100
Umraniye2	150	53	92	−38.7	73.6	90	71	37	−58.9	−47.9	473	396	369	−22	−6.8	409	298	273	−33.3	−8.4	-	-	-	-	-
Average	88.4	92.3	88.6	6.3	−13.4	101.1	82.2	57.6	−15.8	−29.9	282.4	300	255.6	11.8	−8.4	277.5	250	240.7	−0.1	2.5	10.7	22.3	37.4	665.4	540.2
